# Adipokines as Potential Biomarkers in Pregnancy: A Naturalistic Study of Adipokines in Pregnant Women and Newborns

**DOI:** 10.3390/biom15050607

**Published:** 2025-04-22

**Authors:** Cristina Mihaela Ormindean, Răzvan Ciortea, Andrei Mihai Măluțan, Carmen Elena Bucuri, Doru Mihai Diculescu, Cristian Ioan Iuhas, Ciprian Gheorghe Porumb, Vlad Ormindean, Maria Patricia Roman, Ionel Daniel Nati, Viorela Suciu, Alexandru Emil Hăprean, Dan Mihu

**Affiliations:** 2nd Department of Obstetrics and Gynaecology, “Iuliu Hațieganu” University of Medicine and Pharmacy, 400012 Cluj-Napoca, Romania; cristina.mihaela.prodan@gmail.com (C.M.O.); ddiculescu@yahoo.com (D.M.D.);

**Keywords:** maternal obesity, adipokines, pregnancy, fetal development, leptin, adiponectin, metabolic health

## Abstract

Maternal obesity is an escalating public health concern that adversely affects pregnancy outcomes. Adipokines play a key role in regulating metabolism and fetal development, but their dynamic changes during pregnancy remain inadequately understood. **Objective:** This study investigates maternal and fetal adipokine variations throughout pregnancy and their associations with maternal body mass index (BMI), abdominal wall thickness, and neonatal outcomes. **Methods:** A prospective case-control study was conducted involving 74 pregnant women categorized by BMI. Maternal blood samples were collected at mid-pregnancy and delivery, and additional analysis of umbilical-cord blood was performed. Clinical parameters such as BMI, abdominal wall thickness, and fetal growth metrics were also recorded. **Results:** Adiponectin levels were significantly lower in obese pregnancies, whereas leptin and visfatin levels increased with higher maternal BMI. Umbilical-cord blood leptin levels correlated positively with maternal BMI and neonatal birth weight, while ghrelin levels were reduced in neonates born to obese mothers. Significant adipokine fluctuations were observed between mid-pregnancy and delivery. **Conclusions:** Maternal obesity is associated with distinct alterations in adipokine profiles. These findings highlight the potential of maternal adipokines, given their links to maternal adiposity, as predictive biomarkers for adverse pregnancy outcomes and long-term metabolic risks in offspring. Further interventional research is warranted to evaluate targeted strategies aimed at improving perinatal metabolic health.

## 1. Introduction

Obesity during pregnancy has become a significant public health concern and a growing challenge for obstetrics and gynecology specialists. It is associated with increased risks of gestational diabetes, preeclampsia and both fetal and neonatal complications [1,2]. The physiological adaptations that normally occur during pregnancy are profoundly altered in individuals with obesity, resulting in complex endocrine and metabolic dysregulation [3]. Adipose tissue, beyond its role as an energy reservoir, is now recognized as an active endocrine organ. It secretes a variety of adipokines—bioactive peptides and proteins that play critical roles in regulating insulin sensitivity, inflammation, and energy homeostasis [4]. These signaling molecules exert pleiotropic effects on metabolism, immune function, and vascular homeostasis through autocrine, paracrine, and endocrine mechanisms. In the context of obesity, altered adipokine profiles contribute significantly to systemic inflammation and metabolic disturbances, affecting both maternal and fetal health [5,6]. Among adipokines, adiponectin and leptin are the most extensively studied, but newer molecules such as visfatin and ghrelin have attracted attention in recent years due to their diverse and often opposing effects on maternal and fetal health.

Adiponectin, known for its insulin-sensitizing and anti-inflammatory properties, often evinces low concentrations in obesity, potentially exacerbating metabolic disorders during gestation [7]. In contrast, leptin, which regulates satiety and energy expenditure, is often elevated in obese individuals, contributing to leptin resistance and further metabolic disruption [6]. More recently, visfatin has attracted attention due to its insulin-mimetic and pro-inflammatory properties, with emerging relevance in pregnancy-related metabolic disorders. Although ghrelin is not classified as an adipokine, it is a peptide hormone predominantly secreted by the stomach and plays a central role in appetite regulation and energy balance. Despite its different origin, ghrelin was included in this study due to its significant influence on metabolic adaptation during pregnancy and its potential interplay with adipokines in maternal–fetal outcomes [8,9]. These molecules were selected for analysis due to their combined relevance in modulating metabolic and inflammatory responses during gestation, their documented effects relative to alterations in obesity, and their potential as biomarkers in predicting adverse pregnancy and neonatal outcomes 

This study aims to investigate the dynamic changes in circulating levels of adiponectin, visfatin, ghrelin, and leptin in obese pregnant women throughout the gestational period. Specifically, we focus on two key time points: mid-pregnancy (18–22 weeks of amenorrhea) and delivery. Another objective of this study is to investigate the correlations between maternal serum adipokine levels and clinically relevant parameters, including body mass index (BMI), maternal abdominal wall thickness, and cord-blood adipokine concentrations, as well as fetal birth weight. Through this analysis, we seek to analyze the complex interactions between adipokines and maternal–fetal outcomes in the pregnancies of normal-weight, overweight, and obese patients, offering insights into potential biomarkers and therapeutic targets for improving perinatal health.

## 2. Materials and Methods

We conducted a prospective case-control study between January 2021 and December 2022 in which were included patients who were followed in the Obstetrics-Gynecology Clinic II “Dominic Stanca” in Cluj–Napoca. The study sample was calculated based on the establishment of a 95% confidence interval, a 5% margin of error, and a population proportion set at 50%, as recommended in the literature of mathematical statistics for cases in which the results cannot be predicted beforehand. In all, 102 pregnant women were evaluated during the screening period, and the study sample was determined to consist of 81 women. Thus, a total of 81 patients who met the inclusion criteria were enrolled in the study, specifically, pregnant patients between 18 and 22 weeks of amenorrhea and with a BMI ranging between 18.80 kg/m^2^ and 45.72 kg/m^2^ (normal weight, overweight, and obese), after signing the informed-consent documentation. At enrollment, only patients without known associated pathologies were included. However, patients who developed pregnancy complications, such as preeclampsia, during follow-up were retained in the final analysis for comparative purposes. A total of 7 patients were lost from the study due to delivery in other departments or institutions or non-adherence to scheduled follow-up visits during the gestation period, resulting in a final cohort of 74 participants. 

To assess gestational weight progression and to reclassify BMI categories, the baseline utilized was the table in the article “Committee Opinion: Weight gain during pregnancy.” *The American College of Obstetricians and Gynecologists* Number 548 January 2013 (Reaffirmed 2020):

- Total gain in the first trimester of pregnancy: 1.1–4.4 lb = 0.49895–1.9958 kg with a mean value Vm = 1.247375 kg;
- Normal weight: 1 lb = 0.45359 kg/week in the second and third trimesters of pregnancy;
- Overweight: 0.6 lb = 0.27216 kg/week in the second and third trimesters of pregnancy;
- Obese: 0.5 lb = 0.22680 kg/week in the second and third trimesters of pregnancy.

Patients were categorized, based on pre-pregnancy BMI (BMI at conception), as underweight, normal weight, overweight, and obese, as calculated against the weight at that time, W_0_.

We calculated the number of days of pregnancy at the midterm visit and at delivery for each individual pregnant woman: *x* intermediary = *x*_i_ and *x* at delivery = *x*_d_.

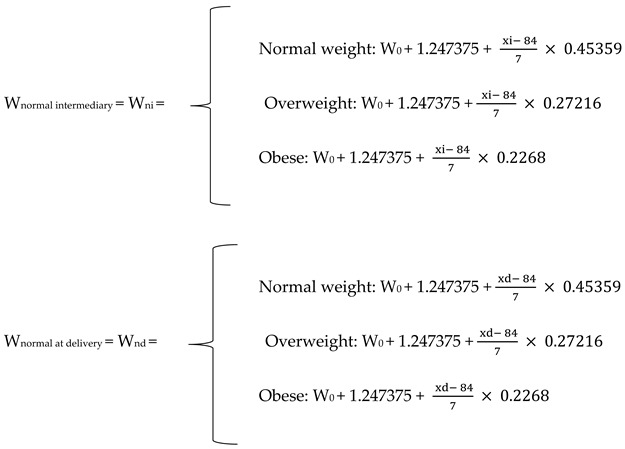

where 84 represents the number of days in the first trimester of pregnancy.

The algorithm for the midterm and time of delivery consultations is as follows:

- If W_measured_ is ≤theoretical normal weight, the patient remains in the same BMI category as at W_0_;
- If W_measured_ is ≥theoretical normal weight, BMI is recalculated and the patient is reclassified accordingly.

Exclusion criteria included pre-existing hypertension or diabetes mellitus, patients with gestational ages outside the defined range (18–22 weeks), those refusing to participate in the study, and patients with multifetal pregnancies. Enrolled patients had blood samples collected to determine adipokine levels at study enrollment (18–20 weeks of amenorrhea) and at delivery; body mass index (BMI) calculated; abdominal wall thickness measured at the white line at approximately 2 cm supraumbilical, both in inspiration and expiration, at study enrollment; and fetal biometry ultrasonographic assessment and fetal growth and development assessment. The biometric data and abdominal wall thicknesses were assessed using the Toshiba Aplio 300. Processing of blood samples for the determination of adipokine levels was performed in the Physiology Department of the “Iuliu Hațieganu” University of Medicine and Pharmacy Cluj–Napoca, using Elisa kits. The study was conducted in accordance with the Declaration of Helsinki and approved by Ethics Commission of the University of Medicine and Pharmacy “Iuliu Hațieganu” Cluj–Napoca (56/22 January 2021).

### Statistical Analysis

The statistical analysis employed descriptive statistical methods, including percentages, means, and standard deviations, for both categorical and continuous variables. For comparative analysis between groups based on continuous variables, *t*-tests assuming equal variances were utilized. Additionally, the correlation between continuous variables was assessed using Pearson’s correlation coefficient. The threshold for statistical significance was established at 0.05. The analyses were conducted using SPSS Statistics Version 30.

## 3. Results

The study cohort consisted of 74 pregnant women whose ages ranged from 20 to 44 years, with a mean age of 31.32 years (SD = 5.38). This relatively broad age distribution reflects a mature reproductive age group, a class which can be associated with varying obstetric outcomes. In terms of parity, the distribution was fairly balanced: 24 women (32.43%) were nulliparous, 29 (39.19%) were primiparous, and 21 (28.38%) were multiparous (Table 1). Classification on the basis of education showed that the majority of the women were in the group associated with higher education (i.e., secondary school 8.11%, vocational school 6.76%, high school 29.73%, post-secondary school 6.76%, and higher education 48.65%). Regarding the living environment, 62.16% of the pregnant women lived in urban areas and 37.84% in rural areas. All pregnancies were singleton pregnancies, and 48.65% were vaginal births and 51.35% by cesarean section. The weight of the newborns ranged from 1750 g to 4800 g, with a mean value of 3277.77 g (SD = 588.96 g). A proportion of 47.30% of the newborns had an initial APGAR score of 10, and 66.22% had a final APGAR score of 10.

BMI categories were defined at three critical time points: pre-pregnancy (before gestation), at 18–22 weeks of amenorrhea, and at birth. Initially, before pregnancy, more than half of the participants (54.05%) were classified as having a healthy weight (normal weight), while 29.73% were overweight, and 16.22% were obese. This baseline classification was based on pre-pregnancy BMI values, in keeping with standard definitions.

During the mid-pregnancy period (18–22 weeks of amenorrhea), the proportions changed slightly: 52.70% of the women remained in the healthy weight category, 27.03% were classified as overweight, and the percentage of those classified as obese increased to 20.27%. By the time of delivery, while the proportion of healthy weight women remained stable (52.70%), a striking shift was observed in the distributions of the higher BMI categories. The proportion of overweight women decreased to 13.51%, whereas the prevalence of obesity surged to 33.78%.

This temporal trend in BMI categorization likely reflects the pattern of gestational weight gain relative to the Institute of Medicine (IOM) recommendations. In many cases, the actual weight gain during pregnancy exceeded the theoretical normal weight-gain range expected for the pre-pregnancy BMI group. Consequently, although initial groupings were made based on pre-pregnancy weight, a subset of women experienced weight gains that resulted in a reclassification to the obese category by the time of delivery. This finding underscores the importance of monitoring gestational weight gain rigorously, as deviations from the recommended ranges may have implications for both maternal metabolic health and fetal outcomes (Table 2).

In addition to anthropometric data, the study also documented a range of comorbid conditions and obstetric complications. Although most comorbidities were infrequent—each affecting 1.35% to 2.70% of the cohort—a few conditions were notable for their higher prevalence or clinical significance:

- Postpartum Bleeding: This complication was observed in 13 women (17.57%), representing the most common adverse event in the cohort. It is important to note this higher incidence, as it may be related to variations in gestational weight gain or other metabolic factors.
- Preeclampsia and Intrauterine Growth Restriction (IUGR): Each of these conditions was noted in four women (5.41%), indicating that hypertensive disorders and fetal growth issues were present in a measurable proportion of the study group.
- Other conditions, including diabetes mellitus (4.05%), hypertension (2.70%), and isolated instances of extrasystolic arrhythmia, cervical dysplasia, uterine fibroids, and surgical history for endometriosis, were recorded at low frequencies. These conditions, despite their low prevalence, are important to report as potential confounders in studies examining metabolic parameters and inflammatory markers such as adipokines.

Collectively, the presence of these comorbidities and complications provides critical context for interpreting the biochemical and anthropometric findings. For example, even though only a minority of women had overt metabolic or inflammatory disorders, the notable increase in obesity by delivery may contribute to altered adipokine profiles, as well as to the increased risk of complications, like postpartum bleeding and preeclampsia, observed in the cohort (Table 3). 

The comparison of adipokines, for both pregnant women and newborns, was performed by classifying the study subjects according to different parameters: BMI of pregnant women at 18–22 weeks of amenorrhea and BMI at birth, and fetal development at 18–22 weeks of amenorrhea and at birth (Appropriate for Gestational Age—AGA/Small for Gestational Age—SGA), respectively.

### 3.1. Comparison of Adipokine Values Using the BMI of Pregnant Women as a Classification Factor

When comparing the groups of pregnant women categorized in terms of BMI at 18–22 weeks at both the intermediary visit and delivery, normal-weight women consistently had higher adiponectin levels, compared to both overweight and obese women. This reduction in adiponectin with increasing BMI is consistent with the literature on obesity-related hypoadiponectinemia. In contrast, leptin levels increased as BMI increased. Normal-weight women had the lowest leptin levels, while overweight and obese groups showed progressively higher levels. The significant differences between the normal-weight group and the other two suggest that elevated leptin is closely associated with higher maternal BMI. Visfatin levels followed a pattern similar to leptin levels, with significantly lower levels in the normal-weight group than in the overweight or obese groups. Again, the lack of a significant difference between overweight and obese groups suggests that visfatin increases when moving from a normal weight status, but the differences plateau or vary less significantly between the overweight and obese categories (Table 4).

In summary, these results indicate a clear trend in which maternal obesity is associated with decreased adiponectin levels and elevated leptin and visfatin levels, both during mid-pregnancy and at delivery. While the normal-weight group significantly differs from the overweight/obese groups, the latter two are similar to each other regarding these adipokine levels. 

For the cord-blood analyses of adipokines, newborns were clustered according to the birth BMI groups of the mothers. The data indicate that umbilical-cord blood adiponectin levels are similar, regardless of whether the mother is of normal weight, overweight, or obese. This suggests that maternal BMI may not influence fetal adiponectin concentrations or that compensatory mechanisms could be maintaining steady levels in the fetal circulation. Umbilical-cord leptin levels are elevated when mothers are either overweight or obese compared to their normal weight. The similarity between the overweight and obese groups suggests that after a certain threshold, further increases in maternal BMI may not lead to additional increases in fetal leptin levels. Elevated cord-blood leptin could reflect increased adiposity or altered metabolic signaling in fetuses of mothers with higher BMI (Table 5).

Ghrelin levels in umbilical-cord blood are lower in infants born to both overweight and obese mothers, compared to those from normal-weight mothers. The lack of additional differences between the overweight and obese groups indicates that the decrease in cord ghrelin may reach a plateau once maternal BMI exceeds normal ranges. Ghrelin plays a role in appetite regulation and energy balance, and lower levels in the cord blood might be indicative of altered fetal energy homeostasis in pregnancies affected by maternal overweight or obesity (Table 6).

### 3.2. Comparison of Adipokine Values According to Fetal and Newborn Development (LGA/AGA/SGA)

Analysis of maternal blood adipokines was also performed by comparing the mean values corresponding to groups of pregnant women classified on the basis of fetal development at the 18–22 weeks of amenorrhea consultation and the development of the newborns at the time of delivery. In the maternal blood at 18–22 weeks of amenorrhea, adiponectin was highest in pregnancies with fetuses classified using biometry as Small for Gestational Age (SGA) (*p* < 0.01). The highest values for leptin were observed in pregnant women with fetuses classified on the basis of biometry as Appropriate for Gestational Age (AGA) (*p* < 0.05), but for visfatin, the mean maternal blood values of AGA fetuses were higher than those of SGA fetuses, without this difference reaching statistical significance. At birth, the fetal development comprised three categories, namely, Large for Gestational Age (LGA), Appropriate for Gestational Age (AGA), and Small for Gestational Age (SGA), with the adiponectin levels in the group of pregnancies with SGA newborns being the highest, followed by the values associated with mothers with LGA and AGA newborns, respectively. The highest value recorded for leptin was recorded in mothers with LGA newborns; this was followed by, in decreasing order, values for mothers with AGA newborns and those with SGA newborns. Visfatin levels reached their highest value in mothers with AGA newborns, followed by mothers with LGA and SGA newborns, respectively. However, statistical significance is not reached in any of these distinctions relating to adipokines (Table 7).

When analyzing the adipokines in cord blood, and classifying newborns according to their development (LGA, AGA, and SGA), we observe that for mean values of adiponectin the differences are minimal and statistically insignificant. This is not the same in the analyses of leptin. The mean value of leptin for LGA newborns is the highest, followed in descending order by the corresponding values for AGA and SGA, reaching statistical significance (*p* < 0.05). For ghrelin the mean value for SGA newborns is the highest, followed by the values for LGA and AGA newborns, respectively, but without reaching statistical significance (Table 8).

### 3.3. Analysis of Abdominal Wall Thickness (AWT) in Pregnant Women

The analysis of the abdominal wall thickness of pregnant women was performed by classifying pregnant women according to BMI at 18–22 weeks of amenorrhea and according to fetal developmental AGA/SGA.

In the analysis based on the BMI classification of pregnancies at 18–22 weeks of amenorrhea, the progressive increase in AWT during inspiration from normal weight (1.61 cm) through overweight (2.20 cm) to obese women (2.80 cm) demonstrates a clear correlation between higher BMI and greater abdominal wall thickness. This suggests that increased maternal adiposity—reflected as thicker subcutaneous fat layers—manifests early in mid-pregnancy. Similarly to the inspiratory measurements, the AWT during expiration increases significantly with higher BMI—from 1.83 cm in normal-weight women, to 2.55 cm in overweight women, and reaching 3.07 cm in obese women. These findings further reinforce that maternal adiposity is associated with greater abdominal wall thickness regardless of the respiratory phase. The slight differences in magnitude between inspiration and expiration likely reflect the dynamic changes in body dimensions during breathing, yet the consistent trend confirms the robustness of AWT as an indicator of maternal adiposity (Table 9).

In the analysis based on the classification of pregnancies according to the grade of fetal development, it was observed that, both in inspiration and expiration, the abdominal wall thickness was greater in pregnancies with AGA fetuses, compared to those with SGA fetuses (*p* < 0.05). This finding may reflect better maternal nutritional status or higher levels of maternal adiposity in women whose fetuses are growing appropriately. A thicker abdominal wall is often an indicator of greater subcutaneous fat deposition, which might correlate with the ability to provide energy stores and nutrients adequate for optimal fetal growth (Table 10).

### 3.4. Correlations

The Pearson correlation coefficient between maternal abdominal wall thickness in inspiration and expiration and adipokine values was calculated, classifying pregnant women according to BMI at 18–22 weeks of amenorrhea. Based on the coefficients obtained, the correlations are weak and very weak. In normal-weight mothers, the correlations between AWT and adipokine levels are generally negligible for adiponectin and leptin, with a slight tendency for higher AWT to be associated with lower visfatin levels (r = −0.197). This suggests that, among women with a normal BMI, differences in abdominal subcutaneous fat are not strongly predictive of changes in circulating adipokine levels. In the overweight group, the relationships are also weak. There is a modest positive trend for leptin (r = 0.114) and visfatin during inspiration (r = 0.156), but these associations become almost negligible during expiration. Overall, in overweight mothers, abdominal wall thickness does not appear to have a strong linear association with these adipokines, although slight trends toward higher leptin (r = 0.229) and visfatin (r = −0.224) with increased AWT are observed during inspiration. Among obese mothers, the pattern is more consistent: increased abdominal wall thickness is associated with lower adiponectin and visfatin levels and with higher leptin levels. Although these correlations are still generally weak, the association for visfatin during expiration is somewhat stronger (r = −0.299), which may imply that in obese pregnancies, greater subcutaneous fat (as measured by AWT) is more closely linked with reduced visfatin levels. The weak positive correlation for leptin suggests that higher maternal adiposity—as reflected by a thicker abdominal wall—is associated with higher leptin levels, a finding that aligns with the established understanding of leptin physiology in obesity.

Analysis of the degree of correlation between abdominal wall thickness at inspiration and expiration, classifying pregnancies based on fetal development determined by fetal biometry (AGA/SGA), showed higher coefficients for pregnancies with fetuses with development corresponding to the gestational moment (AGA), with moderate correlations for adiponectin and weak correlations for leptin and visfatin. For pregnancies with gestationally underdeveloped fetuses (SGA) the correlations were weak for adiponectin and leptin and very weak for visfatin. Interestingly, these coefficients were negative for adiponectin in both the AGA and SGA groups, and for leptin in the SGA group (Table 10).

On the other hand, analyzing the degrees of correlation between maternal blood adipokines and those in the umbilical cord, the data reveal variability in the relationship between maternal and cord-blood adiponectin across BMI groups. In normal-weight and obese mothers, there is a clear positive association, suggesting that maternal adiponectin may influence or be reflective of fetal levels. However, in the overweight group, the near-zero correlation indicates that this relationship is absent or obscured, warranting further investigation into the underlying factors affecting adiponectin transfer or regulation in overweight pregnancies. Across all maternal BMI categories, the strong correlation coefficients (ranging from 0.723 to 0.781) indicate that maternal leptin levels are closely associated with cord-blood leptin levels. This suggests that factors regulating leptin secretion and its placental transfer are maintained across different maternal weight profiles. Consequently, leptin appears to be a robust marker that reflects maternal metabolic status in the fetal circulation, independent of whether the mother is of normal weight, overweight, or obese (Table 11).

Analysis of the correlation between the maternal and umbilical-cord adipokines adiponectin and leptin, categorizing mothers and newborns according to developmental stage at birth, showed that for adiponectin there were strong correlations in LGA and SGA newborns and a moderate correlation in AGA newborns. In contrast, for leptin, the correlation is very strong in LGA and AGA newborns and strong in SGA newborns (Table 12).

## 4. Discussion

This study aimed to elucidate the dynamic changes in maternal and cord-blood levels of adipokines—adiponectin, leptin, and visfatin—and ghrelin across pregnancy, and to explore their associations with maternal body mass index (BMI), abdominal wall thickness (AWT), and fetal growth parameters. Our findings highlight a strong link between maternal adiposity and unfavorable adipokine profiles which may in turn influence fetal metabolic adaptations.

Our analyses confirmed that maternal obesity is associated with a significant reduction in adiponectin levels and elevated leptin and visfatin levels at both mid-pregnancy (18–22 weeks of amenorrhea) and at delivery [2,7,10,11]. Consistent with the literature, the lower adiponectin levels observed in overweight and obese women likely contribute to insulin resistance and an amplified inflammatory state [7,10,11]. In contrast, the progressive increase in maternal leptin levels with higher BMI supports the concept of leptin resistance [12,13,14], wherein elevated circulating leptin may be an adaptive but ultimately ineffective response to increased adiposity. Similarly, visfatin—a marker with insulin-mimetic and pro-inflammatory properties [15,16]—was significantly higher in overweight and obese subjects compared to normal-weight women, though the differences between overweight and obese groups were less pronounced. Interestingly, while overall adipokine patterns remained consistent from mid-pregnancy to delivery, we detected subtle but non-significant trends suggesting that pre-pregnancy BMI exerts a more dominant influence on adipokine secretion than do intra-gestational metabolic adaptations.

The umbilical cord is a pivotal structure in fetal development, serving as an essential mediator of protein transport and regulation. Variations in umbilical-cord proteins, particularly adipokines and growth factors, not only mirror the maternal metabolic environment but also play an active role in shaping neonatal characteristics and future health. Monitoring these modifications can provide valuable biomarkers for assessing neonatal risk and guiding early therapeutic interventions [17,18,19]. Umbilical-cord blood analyses revealed that fetal adiponectin levels remain relatively constant across maternal BMI groups. This finding suggests that mechanisms maintaining fetal adiponectin concentrations may buffer the fetus against maternal metabolic variations [20,21]. In contrast, cord-blood leptin levels were significantly elevated in infants born to overweight and obese mothers, compared to those born to normal-weight mothers, reflecting maternal metabolic status and increased adiposity [20,21]. Moreover, ghrelin levels in the cord blood decreased with rising maternal BMI—an observation that may indicate altered fetal energy balance regulation in the context of maternal obesity [22,23]. Notably, our correlation analyses between maternal and cord-blood adipokine levels demonstrated robust associations for leptin across all BMI groups, whereas the relationship for adiponectin was variable; strong correlations were present in normal-weight and obese mothers but were nearly absent in overweight mothers. Such differential patterns may reflect distinct regulatory mechanisms governing the placental transfer of these hormones.

Our study also assessed maternal AWT as a surrogate marker of adiposity [24,25]. As expected, AWT measured both during inspiration and expiration increased significantly with higher BMI. Importantly, when stratified by fetal growth status, AWT was significantly greater in pregnancies with Appropriate for Gestational Age (AGA) fetuses, compared to those with Small for Gestational Age (SGA) fetuses. This suggests that a thicker maternal abdominal wall, indicative of greater subcutaneous fat reserves, may favor the provision of nutrient levels adequate for fetal growth. However, correlation analyses between AWT and adipokine levels revealed only weak associations overall—with stronger negative correlations between AWT and visfatin observed in obese mothers. These findings imply that while AWT is a reliable marker of maternal adiposity [24,25], its direct influence on adipokine secretion is complex and may be modulated by other metabolic factors [26].

The observed alterations in maternal adipokines, together with the strong associations between maternal and cord-blood leptin, support the hypothesis that maternal metabolic status directly impacts fetal endocrine function [27]. Elevated fetal leptin in the context of maternal obesity may contribute to early programming of adiposity and metabolic risk in offspring [3,20,28,29]. Conversely, the apparent stability of fetal adiponectin despite variations in maternal levels suggests potential compensatory regulatory processes that preserve fetal insulin sensitivity and anti-inflammatory capacity [30,31].

Moreover, the inverse association between maternal AWT and adiponectin—as well as the direct relationship between AWT and leptin—indicates that increased maternal adiposity not only shifts circulating hormone levels but may also contribute to a fetal environment predisposed to metabolic dysregulation [32,33]. These findings stress the importance of monitoring gestational weight gain in accordance with established Institute of Medicine (IOM) guidelines, as deviations may have long-term implications for both maternal health and offspring health [34].

A major strength of this study lies in its comprehensive approach, simultaneously evaluating multiple adipokines alongside objective measures of maternal adiposity (i.e., AWT) and fetal growth metrics. Inclusion of paired maternal and cord-blood samples provided novel insights into maternal–fetal endocrine interactions. Nonetheless, our study has limitations. The relatively modest sample size may constrain the generalizability of our findings, and the observational design precludes the establishment of causality. Additionally, potential confounders such as dietary intake, physical activity, and genetic predispositions were not controlled for and warrant consideration in future research.

Future studies should address these limitations and explore whether interventions targeting maternal adipokine profiles—by means of nutritional or lifestyle modifications—can favorably modulate fetal metabolic programming and reduce the long-term risks of metabolic diseases. Longitudinal follow-up of offspring may elucidate the persistence of these intrauterine hormone alterations and their impacts on childhood and adult metabolic health.

## 5. Conclusions

In summary, our findings demonstrate that maternal obesity is associated with significant alterations in adipokine profiles, as reflected in decreased adiponectin and increased leptin and visfatin levels. These changes are paralleled by altered cord-blood leptin and ghrelin levels and are accompanied by greater maternal abdominal wall thickness, underscoring the complex interplay between maternal adiposity and the fetal metabolic environment. These results reinforce the potential of maternal adipokines—and their association with indices of adiposity—as biomarkers for predicting adverse pregnancy outcomes and long-term metabolic risks in offspring. Future interventional studies are warranted to assess the efficacy of targeted strategies in improving perinatal metabolic health.

## Figures and Tables

**Table 1:** Description of the study population, taking into account age and parity status of pregnant patients.

Number of Pregnant Women (*n*)	74
Age (years)	
range	20–44
average	31.32
SD	5.38
Parity	
nulliparous	24 (32.43%)
primiparous	29 (39.19%)
multiparous	21 (28.38%)

Values in the table are presented as mean (SD).

**Table 2:** BMI changes throughout pregnancy.

	BMI Category		
	Healthy Weight *n* (%)	Overweight *n* (%)	Obesity *n* (%)
Before pregnancy	40 (54.05%)	22 (29.73%)	12 (16.22%)
At 18–22 weeks of gestation	39 (52.70%)	20 (27.03%)	15 (20.27%)
At birth	39 (52.70%)	10 (13.51%)	25 (33.78%)

**Table 3:** Occurrence of pathologies and complications during pregnancy period.

Comorbidities	*n* (%)
Extrasystolic arrhythmia	1 (1.35%)
Cervical dysplasia	1 (1.35%)
Gestational diabetes mellitus	3 (4.05%)
Endometriosis	1 (1.35%)
Uterine fibroids	1 (1.35%)
Hypertension	2 (2.70%)
Breech presentation delivery	1 (1.35%)
Placenta praevia with haemorrhage	1 (1.35%)
Rheumatoid polyarthritis	1 (1.35%)
Preeclampsia	4 (5.41%)
Intrauterine growth restriction	4 (5.41%)
Postpartum bleeding	13 (17.57%)
In vitro fertilization (IVF)	2 (2.70%)
Prolonged pregnancy	3 (4.05%)
Thrombophilia	2 (2.70%)
Bicornuate uterus	1 (1.35%)
Scar tissue in uterus	5 (6.76%)

**Table 4:** Comparison of blood adipokine values in BMI-classified pregnant women at 18–22 weeks of amenorrhea and at delivery.

Adipokine Comparisons per BMI Categories									
Intermediary Visit 18–22 Weeks									
	Normal Weight	Overweight	*p*	Normal Weight	Obese	*p*	Overweight	Obese	*p*
Adiponectin	4.47 (1.19)	3.12 (0.74)	<0.001	4.47 (1.19)	3.08 (0.97)	<0.001	3.12 (0.74)	3.08 (0.97)	NS
Leptin	550.06 (159.78)	675.98 (175.71)	<0.01	550.06 (159.78)	704.97 (91.55)	<0.001	675.98 (175.71)	704.97 (91.55)	NS
Visfatin	10.34 (3.77)	20.88 (9.76)	<0.001	10.34 (3.77)	16.82 (4.99)	<0.001	20.88 (9.76)	16.82 (4.99)	NS
At delivery									
	Normal Weight	Overweight	*p*	Normal Weight	Obese	*p*	Overweight	Obese	*p*
Adiponectin	3.64 (1.18)	2.51 (0.79)	<0.01	3.64 (1.18)	2.53 (1.02)	<0.001	2.51 (0.79)	2.53 (1.02)	NS
Leptin	653.98 (147.12)	810.86 (120.79)	<0.01	653.98 (147.12)	855.30 (122.70)	<0.001	810.86 (120.79)	855.30 (122.70)	NS
Visfatin	7.09 (6.48)	14.52 (4.62)	<0.001	7.09 (6.48)	13.68 (8.41)	<0.001	14.52 (4.62)	13.68 (8.41)	NS

Values in the table are presented as mean (SD).

**Table 5:** Comparison of adipokine levels in umbilical-cord blood, classified by maternal BMI category.

Adipokine Comparison in Umbilical-Cord Blood, Classified by Maternal BMI Category									
	Normal Weight	Overweight	*p*	Normal Weight	Obese	*p*	Overweight	Obese	*p*
Adiponectin	3.37 (0.85)	3.30 (0.71)	NS	3.37 (0.85)	3.34 (0.72)	NS	3.30 (0.71)	3.34 (0.72)	NS
Leptin	546.19 (107.39)	667.01 (104.26)	<0.01	546.19 (107.39)	660.28 (94.36)	<0.001	667.01 (104.26)	660.28 (94.36)	NS

Values in the table are presented as mean (SD).

**Table 6:** Comparison of ghrelin levels in umbilical-cord blood, classified by maternal BMI category.

Ghrelin Comparison in Umbilical-Cord Blood, Classified by Maternal BMI Category									
	Normal Weight	Overweight	*p*	Normal Weight	Obese	*p*	Overweight	Obese	*p*
Ghrelin	2.52 (0.79)	1.78 (0.69)	<0.01	2.52 (0.79)	1.51 (0.52)	<0.001	1.78 (0.69)	1.51 (0.52)	NS

Values in the table are presented as mean (SD).

**Table 7:** Comparison of maternal blood adipokines at 18–22 weeks of amenorrhea and at delivery, classifying pregnant women according to the degree of fetal/newborn development.

	Maternal Adipokines Based on Fetal Development at 18–22 Weeks and at Delivery (Mean, SD)			
	LGA	AGA	SGA	*p*
At 18–22 weeks of gestation				
Adiponectin		3.61 (1.09)	4.48 (1.46)	<0.01
Leptin		637.99 (164.08)	545.50 (160.34)	<0.05
Visfatin		15.20 (8.04)	12.35 (5.95)	NS
At delivery				
Adiponectin	3.37 (1.17)	3.00 (1.19)	3.59 (1.29)	NS
Leptin	875.67 (245.58)	742.47 (152.88)	674.67 (147.74)	NS
Visfatin	8.33 (7.68)	11.23 (8.01)	6.71 (5.00)	NS

Values in the table are presented as mean (SD).

**Table 8:** Comparison of adipokines and ghrelin in umbilical-cord blood, classifying newborns on the basis of their developmental stage.

	Umbilical-Cord Adipokines and Ghrelin (Mean, SD)			
	LGA	AGA	SGA	*p*
Umbilical-cord blood				
Adiponectin	3.61 (0.58)	3.31 (0.76)	3.42 (1.00)	NS
Leptin	703.60 (143.26)	601.25 (106.35)	544.17 (127.94)	<0.05
Ghrelin	2.16 (1.07)	1.97 (0.81)	2.63 (0.67)	NS

Values in the table are presented as mean (SD).

**Table 9:** Analysis of maternal abdominal wall thickness, based on BMI groups, at 18–22 weeks of amenorrhea.

Abdominal Wall Thickness at 18–22 Weeks (cm)									
	Normal Weight	Overweight	*p*	Normal Weight	Obese	*p*	Overweight	Obese	*p*
During inspiration	1.61 (0.46)	2.20 (0.47)	<0.001	1.61 (0.46)	2.80 (0.48)	<0.001	2.20 (0.47)	2.80 (0.48)	<0.001
During expiration	1.83 (0.55)	2.55 (0.52)	<0.001	1.83 (0.55)	3.07 (0.47)	<0.001	2.55 (0.52)	3.07 (0.47)	<0.01

Values in the table are presented as mean (SD).

**Table 10:** Analysis of maternal abdominal wall thickness, based on fetal developmental groups AGA/SGA.

	Abdominal Wall Thickness (Mean, SD)		
	Mothers with Fetuses		
	AGA	SGA	*p*
Abdominal wall thickness during inspiration	2.09 (0.68)	1.77 (0.52)	<0.05
Abdominal wall thickness during expiration	2.37 (0.75)	1.99 (0.58)	<0.05

Values in the table are presented as mean (SD).

**Table 11:** Pearson’s correlation coefficients between maternal and cord-blood adipokines, categorizing mothers and newborns according to maternal BMI.

	Maternal Adiponectin		
	Normal Weight	Overweight	Obese
Cord-blood adiponectin	0.556	0.028	0.670
	Maternal leptin		
	Normal Weight	Overweight	Obese
Cord-blood leptin	0.781	0.728	0.723

**Table 12:** Pearson’s correlation coefficients between maternal and cord-blood adipokines, classifying mothers and newborns according to development at birth.

	Cord-Blood Adiponectin		
	LGA	AGA	SGA
Maternal adiponectin	0.610	0.419	0.767
	Cord-blood leptin		
	LGA	AGA	SGA
Maternal leptin	0.834	0.807	0.781

## Data Availability

The raw data supporting the conclusions of this article will be made available by the authors on request.
